# Ten-year prescription trend of second-generation antipsychotics in adult psychiatric patients from 2014 to 2023 – a territory-wide population-based study

**DOI:** 10.1192/j.eurpsy.2025.552

**Published:** 2025-08-26

**Authors:** S. Pan, B. P. Or, T. M. Li, N. Y. Chan, T. C. Yip, V. W. Wong, Y. K. Wing, J. W. Chan

**Affiliations:** 1 Department of Psychiatry; 2 Department of Medicine and Therapeutics, The Chinese University of Hong Kong, Hong Kong, Hong Kong

## Abstract

**Introduction:**

Second-generation antipsychotics (SGAs) have been increasingly used as off-label treatments for non-psychotic illnesses, there is a lack of updated data to describe its prescription among psychiatric populations.

**Objectives:**

This study aims to describe the most recent ten-year trend of SGA prescriptions and to identify the probable psychiatric diagnoses associated with the prescription among adult psychiatric patients in Hong Kong.

**Methods:**

All patients aged 18 or older who had a psychiatric diagnosis and were initiated on an SGA based on the population-based electronic health record from the Clinical Data Analysis and Reporting System (CDARS) in Hong Kong between 2014 and 2023 were included. The probable psychiatric indication was defined as the diagnosis recorded within 6 months from the date of SGA initiation and was classified into four groups according to the ICD-9-CM categorization of Mental Disorders (290-319). Trends were examined using joinpoint analyses. The prescription trend was re-analyzed using an alternative classification into groups of Schizophrenia (SCZ), Non-schizophrenia serious mental illness (non-SCZ SMI), and Non-SMI.

**Results:**

A total of 79,681 patients (Female 57.9%, Age 56.8±22.5years) were included. There is significant increase in SGA prescriptions in patients with a probable indication within the ICD-9-CM category of ‘Non-psychotic Mental Disorders’ and ‘Organic Psychotic Conditions’, with an average annual percent change (AAPC) of 1.9% (p<0.01) and 1.7% (p<0.01), respectively; while the probable indications under ‘Other Psychoses’ decreased with an AAPC of -2.2% (p<0.01) (Figure 1). Importantly, the prescriptions among patients with probable diagnostic indications under the group of SCZ and non-SCZ SMI decreased with an AAPC of -6.9% (p<0.01) and -0.8% (p=0.03), respectively, while the non-SMI indications rose from 60.3% to 70.0% from 2014-2023 (AAPC=1.7%, p<0.01) (Figure 2).

**Image 1:**

**Figure:**
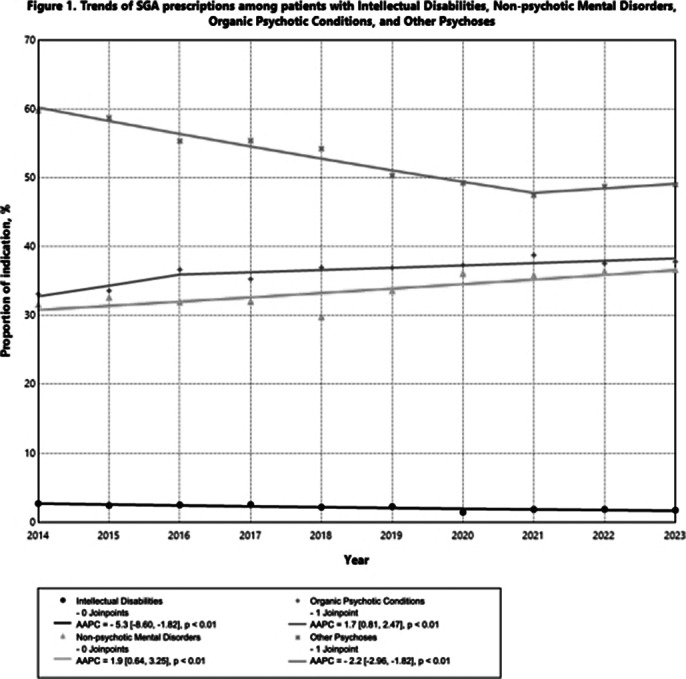

**Image 2:**

**Figure:**
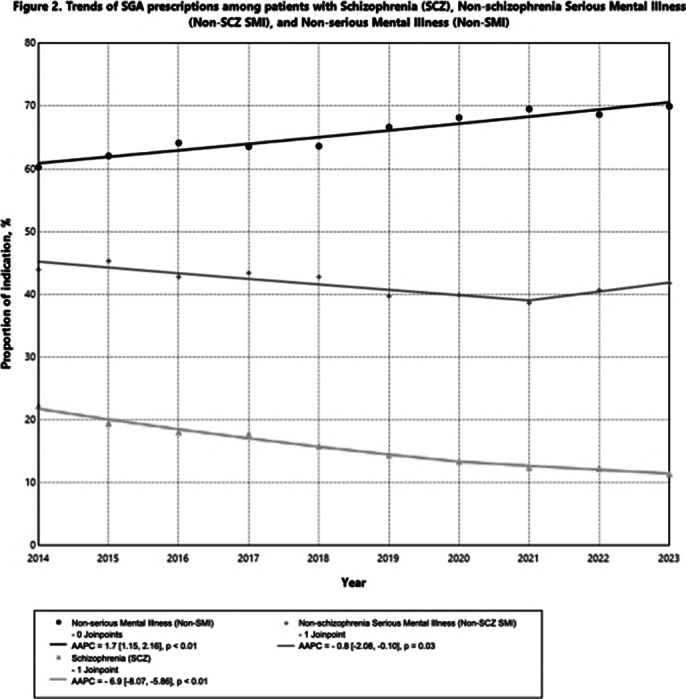

**Conclusions:**

Our study provided important data demonstrating a trend of increasing proportions of SGA prescriptions for non-psychotic and non-SMI indications.

**Disclosure of Interest:**

S. Pan: None Declared, B. Or: None Declared, T. Li: None Declared, N. Y. Chan: None Declared, T. Yip: None Declared, V. Wong Shareolder of: Stock: Co-founder of Illuminatio Medical Technology , Grant / Research support from: Research grants: Gilead Sciences, Consultant of: Consultancy: AbbVie, AstraZeneca, Boehringer Ingelheim, Echosens, Gilead Sciences, Intercept, Inventiva, Merck, Novo Nordisk, Pfizer, Sagimet Biosciences, TARGET PharmaSolutions, Visirna , Speakers bureau of: Lectures: Abbott, AbbVie, Echosens, Gilead Sciences, Novo Nordisk, Unilab, Y. K. Wing Consultant of: YKW received consultation fee from Eisai Co., Ltd., honorarium from Eisai Hong Kong for lecture, travel support from Lundbeck HK limited for overseas conference., J. Chan Speakers bureau of: JWYC received personal fee from Eisai Co., Ltd and travel support from Lundbeck HK limited for overseas conference.

